# Chicken Egg White Gels: Fabrication, Modification, and Applications in Foods and Oral Nutraceutical Delivery

**DOI:** 10.3390/foods13121834

**Published:** 2024-06-11

**Authors:** Junhua Li, Xuechun Wang, Cuihua Chang, Luping Gu, Yujie Su, Yanjun Yang, Dominic Agyei, Qi Han

**Affiliations:** 1 State Key Laboratory of Food Science and Resources, Jiangnan University, Wuxi 214122, China; 2 School of Food Science and Technology, Jiangnan University, Wuxi 214122, China; 3 Collaborative Innovation Center of Food Safety and Quality Control in Jiangsu Province, Jiangnan University, Wuxi 214122, China; 4 Department of Food Science, University of Otago, Dunedin 9054, New Zealand; 5 School of Science, STEM College, RMIT University, Melbourne, VIC 3000, Australia

**Keywords:** chicken egg white, egg white proteins, gelation, hydrogels, nutraceutical delivery

## Abstract

Chicken egg white (EW) proteins possess various useful techno-functionalities, including foaming, gelling or coagulating, and emulsifying. The gelling property is one of the most important functionalities of EW proteins, affecting their versatile applications in the food and pharmaceutical industries. However, it is challenging to develop high-quality gelled foods and innovative nutraceutical supplements using native EW and its proteins. This review describes the gelling properties of EW proteins. It discusses the development and action mechanism of the physical, chemical, and biological methods and exogenous substances used in the modification of EW gels. Two main applications of EW gels, i.e., gelling agents in foods and gel-type carriers for nutraceutical delivery, are systematically summarized and discussed. In addition, the research and technological gaps between modified EW gels and their applications are highlighted. By reviewing the new modification strategies and application trends of EW gels, this paper provides insights into the development of EW gel-derived products with new and functional features.

## 1. Introduction

In the ever-evolving field of food science, the exploration of the design, fabrication, modification, and application of food proteins holds significant relevance. Protein-based ingredients are fundamental components in a wide array of food products, contributing not only to their nutritional profile but also to their sensory attributes and functional properties. Among these proteins, chicken egg white (EW) proteins have garnered significant attention due to their multifaceted techno-functionalities, including gelling or coagulating, foaming, and emulsifying properties [1].

The versatility of EW proteins makes them promising candidates for various applications in the food and pharmaceutical industries. The gelling properties of EW proteins are of particular significance, as they impart a distinctive texture to foods. This property can be exploited to deliver nutrients and flavors by forming a three-dimensional network. In comparison to other animal proteins, egg white proteins are relatively inexpensive and exhibit favorable gel properties in a range of conditions. From enhancing texture and mouthfeel in food products to serving as carriers for oral nutraceutical delivery systems, EW proteins offer a diverse range of functionalities that can be tailored to meet specific application requirements. However, achieving the full potential of EW proteins necessitates a deeper understanding of their molecular structure, gelation mechanism, and modification strategies.

Recent studies, such as those by Zang and Zhang [2], have highlighted that EW gels produced using conventional methods such as heat-, alkali-, ion-, and acid-induced techniques often exhibit relatively weak gel strength. This underscores the need to investigate novel strategies aimed at enhancing the gelling properties of EW proteins. Furthermore, Razi and Fahim [1] conducted a comprehensive review of the gelling, foaming, and emulsifying properties of EW proteins, but their analysis did not specifically delve into the individual functional properties of EW proteins to offer insightful perspectives. In addition to the aforementioned research, the application potential of EW proteins has been explored in contexts such as EW-based bioactive substance delivery systems [3] and EW-based biomaterials for biomedical applications [4,5]. However, there remains a gap in our understanding of how EW gels could be used in product innovation. Therefore, the exploration of novel design, fabrication, and modification strategies is imperative to enhance the gelling properties of EW proteins, thereby unlocking new avenues for their versatile applications.

This review aims to provide a comprehensive overview of the gelling properties exhibited by EW proteins, outline innovative strategies encompassing physical, chemical, and biological methods aimed at enhancing the gelling characteristics of these proteins, and explore the diverse applications of EW proteins as food materials and valuable nutraceutical supplements in the form of EW hydrogels, nanogels, and aerogels. Importantly, it emphasizes the underlying mechanisms and key distinctions associated with various modification techniques that contribute to the enhancement of EW protein gels while shedding light on the existing gaps between modified EW gels and their practical applications within the food industry.

## 2. Gelation of EW Proteins

### 2.1. Roles of EW Proteins in Gel Formation

**Figure 1:**
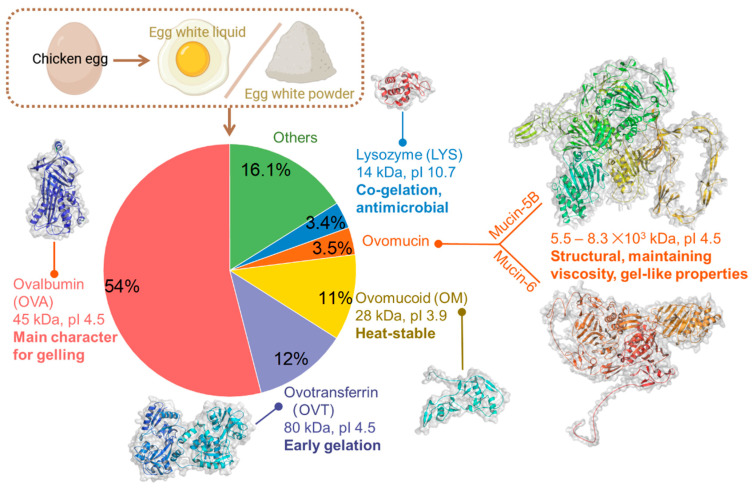
Composition and gelling properties of EW proteins derived from EW liquid or EW powder. The structural information of ovalbumin (1OVA), ovotransferrin (1OVT), and lysozyme (1DPX) was obtained from Protein Data Bank. Ovomucoid and two subunits of Ovomucin were visualized by AlphaFold DB P01005, AF-Q98UI9-F1, and AF-F1NBL0-F1, respectively. Some AlphaFold structural regions may be unstructured in isolation.

Eggs consist of three main components: EW (60%), yolk (30–33%), and eggshell (9–12%). EW is a mixture of water (~88%), proteins (~11%), and carbohydrates (~1%), but the EW proteins are the main gelling substance in EW gels. EW proteins consist of five major proteins, including ovalbumin (OVA, 54% *w*/*w*), ovotransferrin (OVT, 12–13% *w*/*w*), ovomucoid (OM, 11% *w*/*w*), ovomucin (1.5–3.5% *w*/*w*), lysozyme (LYS, 3.4–3.5% *w*/*w*), and other small proteins (Figure 1). OVA is the major globular protein of EW proteins with a molecular weight (MW) of 44.5 kDa, an isoelectric point (pI) of 4.5, and a denaturation temperature (DT) of 84 °C. It plays a leading role in the gelling properties of EW [6]. OVT is the second most important protein affecting the gelation of EW due to its heat sensitivity (DT of 61–65 °C). It is involved in the early stages of EW gelation, together with ovomucin and LYS [7]. OM is a heat-stable glycoprotein (28 kDa), with the heat stability arising from the high carbohydrate content (around 25–30% of OM) [8]. Ovomucin confers gel-like properties and is composed of two subunits with MWs of 5.5–8.3 × 10^3^, including alpha-ovomucin (MUC5B) and beta-ovomucin (MUC6). Notably, LYS could promote the co-gelation of hetero-protein co-aggregate units formed by the electrostatic interactions between OVA and LYS, thus leading to a low critical gelation concentration [9].

Given the diversity of the structures and species of EW proteins, the investigation of specific secondary, tertiary, or quaternary structural changes of an individual EW protein in the EW gel formation is difficult. The gelling of EW depends on the net effects of all the different EW proteins, although OVA plays an important role. The structural changes in EW gels are dependent on their physicochemical properties such as particle size, surface charge, surface hydrophobicity, secondary structural changes, and aggregation [10]. These properties are discussed in the following sections.

### 2.2. Gelation Mechanisms of EW Proteins

**Figure 2:**
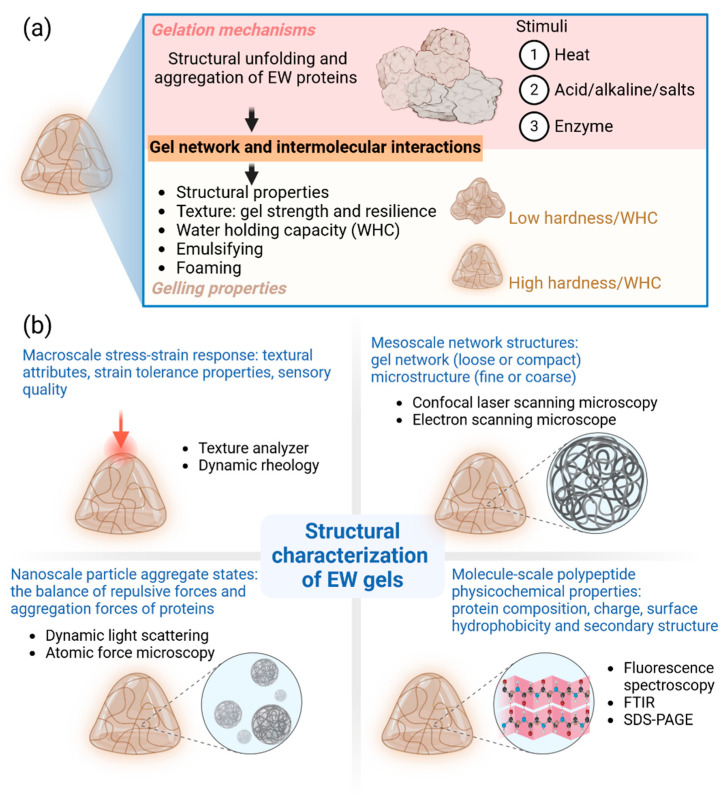
(**a**) The gelation mechanism and gelling properties of EW gels. (**b**) The structural characterization of EW gels in macro-scale, meso-scale, nano-scale, and molecule-scale.

As mentioned earlier, the gelation of EW is a complex process due to the involvement of multiple proteins. Typically, structural unfolding and aggregation of EW proteins drive the gelation process. Moreover, the formation of the gel network and intermolecular interactions within the EW gels are crucial and impact the properties of the gel (Figure 2a). External stress, including heat, acid/alkaline/salts, and enzymes, is the stimulus for EW protein gelation. Heat-induced gelation is the most common method to form EW gels. With increasing heating intensity, the EW proteins can experience conformational changes involving denaturation and unfolding of secondary structures (α-helices and β-sheets), thus exposing hydrophobic groups and leading to protein aggregation through hydrophobic interactions. Hydrophobic interactions are the dominant force involved in the aggregation and gelation of EW proteins, while hydrogen bonds and disulfide bonds play a complementary role [11]. When heat-induced gelation of EW proteins occurs at extremely acidic/alkaline pHs, electrostatic repulsive forces play a bigger role than hydrophobic interactions because most of the proteins assume a net charge that is away from their isoelectric points [12]. In this scenario, EW proteins tend to form a semi-transparent gel, possibly due to the formation of small linear ‘stringy’ gels [13].

The use of microbial transglutaminase (MTGase) is another technique often used to improve the appearance and texture of foods. MTGase works by catalyzing deamidation and crosslinking between protein intra- or inter-chain glutamine (acyl donor) and lysine (acyl acceptor) peptide residues [14]. However, native EW proteins are not good substrates of MTGase due to their compact structures. Alavi and Emam-Djomeh [15] reported that EW proteins can only be effectively cross-linked by MTGase to form a gel network after preheating the EW at 85 °C for 30 min at alkaline conditions (pH 11.3). This pretreatment allows for the exposure of more glutamine and lysine residues on the surface of the protein molecules.

### 2.3. Properties of EW Gels

Extensive investigations have been conducted into the properties of EW gels, including their structural properties, texture (i.e., gel strength and resilience), water holding capacity (WHC), and emulsifying and foaming attributes (Figure 2a) [10,16]. The texture of EW gels not only reflects vital features such as gel hardness/strength and resilience but also exerts an influence on gel digestibility. Notably, our prior study found that low-hardness EW gels are susceptible to in vitro digestion due to weakened hydrogen bond/hydrophobic interactions [10]. Furthermore, Li and Li [17] revealed a positive correlation between WHC and gel strength in EW gels, attributed to the presence of a compact gel network. The acid/heat-induced aggregation of EW proteins exposes more hydrophobic and charged groups on the surface of fibrillar protein aggregates, thereby enhancing interfacial adsorption activity [18]. These interconnected properties and intermolecular interactions collectively contribute to shaping the overall functionality of EW gels.

### 2.4. Structural Characterization of EW Gels

The structural properties of EW gels are usually characterized to understand the properties and the gelling mechanism of EW gels, which involves the characterization of the macro-scale stress-strain response, meso-scale network structure, nano-scale particle aggregate state, and molecule-scale polypeptide physicochemical properties (Figure 2b).

In the characterization of the macro-scale stress-strain response, texture analysis and small-strain dynamic rheology are the two most widely used techniques to provide the textural attributes and strain tolerance properties of EW gels. Moreover, the textural profile analysis could also reflect the sensory qualities of the protein gels. Meso-scale network structures can be imaged by using confocal laser scanning microscopy or electron scanning microscopy. These techniques provide critical information on the gel network (loose or compact) and microstructure (fine or coarse). An EW gel with a fine and compact network is beneficial for applications that rely on water retention [19]. Nano-scale particle aggregate states are affected by the balance of repulsive forces and aggregation forces among proteins and other charged molecules. Nanoscale particle aggregates with different morphologies/sizes exhibit different gelling, emulsifying, and foaming properties, and have significantly different affinities for hydrophobic nutraceuticals [1]. The macro-scale, meso-scale, and nano-scale characteristics of EW gels are all closely correlated with the molecule-scale properties such as protein composition, surface hydrophobicity, and secondary structures. These properties can be characterized by biochemical techniques such as fluorescence spectroscopy, Fourier-transform infrared spectroscopy (FTIR), and sodium dodecyl sulfate–polyacrylamide gel electrophoresis (SDS-PAGE) [20].

## 3. Strategies for Modifying the Gelling Properties of EW Proteins

**Figure 3:**
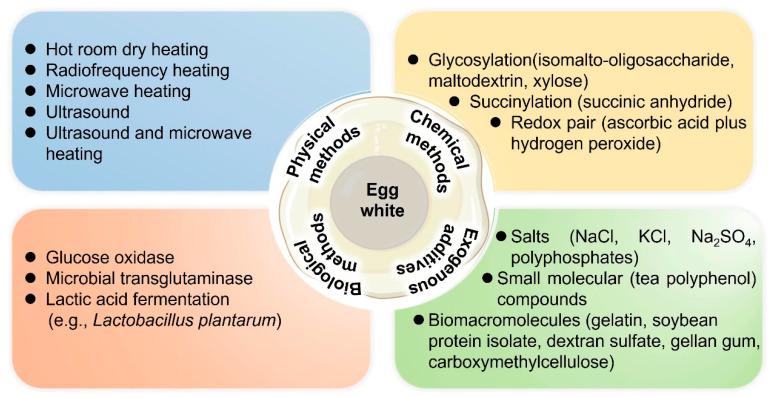
Physical, chemical, biological, and exogenous addition methods for modifying the gelling properties of EW proteins.

To improve the gelling properties of EW proteins, four strategies have been proposed: physical, chemical, biological, and exogenous addition methods. Below, we review the four strategies and summarize their key approaches. Figure 3 shows the main techniques used and Table 1 summarizes the action mechanisms, advantages, and drawbacks of these different methods.

### 3.1. Physical Methods

Heat treatment is one of the most common physical techniques used for improving the gelling properties of EW proteins. Generally, increasing temperature contributes to the gel strength, denaturation, and consequently, enhanced digestibility of EW proteins after in vitro gastro-intestinal digestion [37]. However, overheating can result in excessive protein aggregation and the formation of a compact microstructure, leading to the reduced digestibility of EW gels.

In the egg processing industry, a hot room (HR) is used for traditional thermal pasteurization of EW powder at high temperatures (70–80 °C) for several days [38]. HR dry heating treatment is also an effective method to ameliorate the gelling properties of EW proteins by promoting the formation of ordered secondary structures, particularly β-sheets [39]. In one study, Ma and Chi [40] investigated the effect of dry heating on the hydrothermal aggregation characteristics of EW powder solutions. Their results showed that dry heating hindered the subsequent bulk aggregation of EW powders in aqueous solutions because of the formation of more soluble linear aggregates. The study of Ma and Shan [21] demonstrated that the linear aggregation of dry-heated EW powder occurring on the protein surface was responsible for the improved rheological and gelling behavior. However, traditional HR treatments heat the product from the outside, resulting in a large temperature gradient between hot (outside edges) and cold spots (geometric centers).

Radiofrequency (RF)-assisted thermal processing is a promising thermal processing technology due to its ability to provide rapid heating to achieve target temperatures [41]. In comparison to HR treatment, RF treatment can volumetrically heat the product by vibrating the water molecules and ions that exist throughout the product. Kar and Wei [42] compared the differences in the solubility and gelling properties of EW powder after RF and HR heat treatments. Their results showed that the gel firmness of RF-treated EW powder was 48.6% higher than that of HR-treated EW powder on the 0th d at 80 °C. This indicates that RF treatment could quickly improve the gelling properties of EW powder compared to the traditional HR processing used in the industry, which takes 5–6 days. Interestingly, Kar and Guha [22] also showed in their research that RF processing did not cause significant changes in the secondary conformation of the EW protein structure when compared to HR processing. Therefore, RF processing is a potential technique that could replace HR processing as an alternative to pasteurizing and modifying egg white powder.

Microwave-assisted thermal processing, (or microwave heating (MH)), is another effective method to improve the shelf life and textural quality of food products [43,44]. MH was more beneficial for the formation of firmer EW gels than the corresponding conductive heating, and this observation could be related to the formation of strong non-covalent interactions among EW protein molecules during MH treatment [23]. In addition to improving the gelling properties of EW proteins, MH can improve the foaming performance of EW powder by inducing the structural transition of β-sheets into α-helices [45]. Furthermore, microwave-assisted phosphorylation processing could significantly promote the phosphorylation modification of egg white proteins, thus resulting in improved functional properties [46].

Electron beam irradiation (EBI) is another technology in protein modification that has been attracting interest. In a recent study, EBI-treated EW powders showed higher emulsifying stability indices than the untreated EW powder because of the partial protein denaturation and the formation of flexible structures [47]. In addition, EBI-treated EW powder had improved solubility due to protein depolymerization at high-dose EBI. These findings provide a theoretical basis for the application of EBI technology in the modification of EW powders. However, whether EBI technology can be used to improve the gelling properties of EW powders remains to be studied.

Ultrasound, i.e., sound waves with a frequency above the threshold of human hearing (≥20 kHz), has been extensively applied for the modification of food proteins [48]. Ultrasound can be classified into two categories: low intensity (1 W/cm^2^) with high frequency (5–10 MHz) and high intensity (10–1000 W/cm^2^) with low frequency (20–100 kHz). At present, there is great interest in high-intensity ultrasound because its propagation could induce structural changes in proteins through ultrasonic cavitation. In recent years, ultrasound has been frequently used to improve the gel properties of EW proteins. For example, Jun and Yaoyao [24] studied the effects of different frequencies, temperatures, and durations on the physicochemical and structural properties of EW proteins. Their findings showed that the particle size of ultrasound-treated EW proteins was greatly decreased compared with that of untreated EW proteins, and a low particle size is helpful in increasing the WHC of EW gels. A similar result was observed by Xue and Tu [49], who demonstrated that the hardness and WHC of EW gels can be improved by promoting the cross-linking of proteins to form a dense gel structure after ultrasonic treatment.

In most cases, it is important to use proper ultrasound parameters for the functionality improvement of EW proteins. Deng and Xu [50] found that an EW film prepared by proper high-intensity ultrasound treatment was more flexible and compact than an EW film without extra treatment—a phenomenon that positively correlated to the number of hydrophobic groups and the intermolecular hydrogen bonds. However, after excessive ultrasound treatment, the intermolecular connections between proteins were weakened, and the film became stiffer due to the decrease in the number of hydrophobic groups and intermolecular hydrogen bonds.

In other developments, the synergistic modification of ultrasound and other methods have been proposed to enhance the gel properties of proteins further. Xue and Liu [51] reported that ultrasound and sodium pyrophosphate had synergetic effects in increasing the hardness and cohesiveness and decreasing water mobility of duck EW gels by enhancing hydrophobic and hydrogen bond interactions. Since ultrasound has a strong cavitation effect that produces extreme shear forces, resulting in highly efficient mixing and homogenization, it has been used to improve the inhomogeneity of MH-treated EW powders. Liu and Jin [25] have shown that the EW treated by integrating ultrasound and microwaves had lower apparent viscosity and higher zeta potential values, hydrophobicity, and gel strength than EW treated by traditional heating pasteurization.

### 3.2. Chemical Methods

The Maillard reaction is regarded as one of the most frequent chemical reactions, which has received much attention as a facile, natural, and non-toxic method to improve the technological functionalities of proteins [52]. Heating of protein solutions or dried protein powders in the presence of sugars can result in the formation of covalent protein– sugar complexes via the Maillard reaction, which increase the hydrophilic character of the proteins and change the functional properties [53]. Wang and Li [54] reported that the gelling ability of EW powder can be improved after isomalto-oligosaccharide glycation, which was associated with an increase in hydrogen bonds and hydrophobic interactions. Recent research showed that the glycosylation of EW powder with maltodextrin (MD) led to a more homogenous gel network and an increase in gel hardness and WHC [55]. These results can be ascribed to the transition of β-sheets and the formation of more disulfide bonds. Except for the effect of sugar species, physical processing also affects the glycation rate, as well as the structures and functionalities of glycosylated proteins. Hu and Chen [56] compared the effects of 480 and 640 W of MH and equivalent conventional heating for 5, 10, and 15 min on the properties of OVA–glucose (1:1, *w*/*w*) mixtures. The results showed that MH could accelerate the OVA glycation, particularly at high power levels, and resulted in a higher glycation rate than that of conductive heating, thus significantly improving antioxidant activities and emulsifying capacity. Furthermore, MH-assisted glycosylation modification can also improve the foaming performance of EW powder more significantly than single microwave treatment due to an increase in protein solubility and a decrease in the degree of protein aggregation [57]. Likewise, ultrasound treatment could also enhance the degree of glycation and gel properties of OVA–xylose conjugates through the Maillard reaction, which was due to the unfolding of the protein structure, a higher disulfide bond content, and a compact microstructure [58]. In addition, ball milling has been reported as an efficient and eco-friendly processing method to modify the structural and functional properties of food proteins [59]. Wu and Zhang [60] used ball milling as a pretreatment method to increase the glycosylation of EW proteins. Their study demonstrated that ball milling pretreatment changed the secondary and tertiary structures of the protein, thus prominently enhancing the degree of glycosylation reactions of EW powder and the WHC and springiness of EW powder gels. Wei and Xiao [26] investigated the effects of pH and temperature on the conformational changes of glycosylated EW proteins (D-xylose: proteins = 0.5:1 (*w*/*w*), pH 7.0, 60 °C for 3 h), which modulate its gelling properties. Results showed that alkaline conditions and increasing temperature could promote the expansion of the flexible regions and the flexibility of the protein’s spatial structure. These changes could be beneficial to the inter-crosslinking of protein molecules.

Succinylation is a widely used modification method that can introduce succinyl groups to the free amino group of a protein molecule through a nucleophilic substitution reaction [61]. Hu and Ma [62] investigated the effects of succinylation on the gel behavior of OVA upon heating. The obtained results showed that the acylated OVA hydrogels exhibited higher gel hardness, resilience, and WHC than the native OVA hydrogels. The formation of a uniform and dense network structure strengthened hydrophobic interactions and hydrogen bonding and the substantial transition of α-helices into β-sheets was responsible for the improvement in the gelling capacity of acylated OVA.

Except for heat-set hydrogels, EW proteins can also form cold-set hydrogels through chemical modification. Alavi and Momen [63] developed a new gelation method based on the radical cross-linking of proteins by a non-toxic redox pair consisting of ascorbic acid and hydrogen peroxide. This redox pair modification mainly promoted the unfolding and aggregation or increase in surface hydrophobicity and particle size of EW proteins and the formation of disulfide bonds. At pH 10 and 11, redox-pair-modified EWs had the ability to form cold-set hydrogels.

### 3.3. Biological Modification

In addition to physical and chemical routes, EW proteins can also be modified using biological processes. In a recent study, EW was treated by glucose oxidase (GOD) and yeast to remove the glucose and improve its functionalities [64]. It was observed that the GOD treatment increased the hardness of the EW gel by increasing the surface hydrophobicity (H0) and ordered secondary structures; however, yeast fermentation led to the formation of a poor EW gel with low hardness due to the porous structure brought by increased foam capacity.

Microbial transglutaminase (MTGase) is an important biological method used to modify protein molecules. Alavi and Emam-Djomeh [15] found that the thermally denatured EW proteins made in an alkaline pH can be effectively cross-linked by MTGase in a concentration-dependent manner, thus promoting the formation of a stronger and more elastic three-dimensional network structure. They suggested that the EW cold-set gels treated by MTGase and glucono delta-lactone (GDL) with enhanced mechanical properties have the potential to be used for delivering sensitive compounds and as scaffolds in tissue engineering and biomedical products. Moreover, MTGase was effective in improving the acid-induced cold-set hydrogels prepared by thermal co-aggregates from mixed EW proteins and a hempseed protein isolate by adding GDL [65]. The combination of ultrasound and MTGase was also efficient in preparing EW/gelatin composite cold-set hydrogels with high hardness and storage modulus [27].

Apart from the classical enzyme crosslinker, MTGase, Liu and Li [66] explored a modification method using compound protease and phosphate to improve the functional properties of OVA. This work showed that synergistic modification with compound protease hydrolysis and phosphorylation could effectively improve the solubility, gel strength, and thermal stability of OVA. The hydrolysis of OVA could expose more phosphorylation sites on the protein, which could contribute to forming new products through phosphorylation, resulting in changes in the secondary structure of OVA and the formation of ordered gel structures.

Lactic acid bacteria (LAB) have been widely employed in fermented foods, owing to their enhancement of nutritional value and increase in shelf-life through the fermentation process involving acidification, protein hydrolysis, and production of metabolites. A short-term lactic acid bacteria fermentation method has been proposed to improve the gel properties of liquid whole eggs [67]. The study showed that the surface porous structure of the gel almost disappeared after moderate *Lactiplantibacillus plantarum* fermentation (37 °C, 6–9 h), which is beneficial to the improvement of springiness and water retention. However, the hardness of whole egg gels was decreased, along with a decrease in system pH. Similarly, Chen and Wang [28] found that *Lactiplantibacillus plantarum* fermentation (37 °C for 7 h) led to the decline of EW gel hardness, possibly due to the partial consumption of EW proteins, but promoted the formation of a bright gel appearance by consuming the residual glucose in EW. The above results indicated that lactic acid bacteria fermentation could improve gel qualities other than hardness. Therefore, this novel process of biological modification could facilitate the development of a new generation of egg products with enhanced functionalities.

### 3.4. Exogenous Additives

#### 3.4.1. Salts

The addition of exogenous additives is a simple and efficient method to improve the gelling properties of EW proteins. In many food products, the addition of salt and hydrophilic colloids has a marked impact on the final structure, texture, and stability of protein gels. Salts, such as NaCl and polyphosphates, can modify the network formation and the rheological properties of gels by changing the surface charges, structural state, and inter-molecule forces of EW proteins [68]. The addition of different types of ions is an effective strategy to control the gelling property of EW proteins. Compared with NaCl and KCl, the EW gels with Na_2_SO_4_ showed better texture profiles and elastic moduli, which were related to the formation of large aggregates and strengthened gel networks [29]. In addition, the salt NaCl could promote the formation of a rigid alkaline ovalbumin gel by reducing the electrostatic repulsion of protein molecules and facilitating their aggregation [30].

Phosphates, especially polyphosphates, have been extensively used in processed meat products and dairy products [69,70]. Generally, the effects of phosphates on the gelation of meat proteins are largely attributed to the swell of muscle fibers, the dissolution of myofibrillar protein, and the reinforcement of WHC on account of the increase in pH away from the isoelectric point and ionic strength [71]. Recently, Jin and Chen [19] investigated the impact of phosphates (sodium pyrophosphate, sodium tripolyphosphate, and sodium hexametaphosphate) on the gelling properties of heat-induced EW gels. It was observed that the hardness, springiness, and WHC of the gels treated with sodium pyrophosphate and sodium tripolyphosphate were significantly improved compared to the control group and sodium hexametaphosphate group because of the formation of more uniform and denser network structures with higher disulfide bond contents. In comparison to neutral salt, alkaline polyphosphates upregulate both pH values and ionic strength, as well as having intricate impacts on the performance of the EW hydrogel.

#### 3.4.2. Small Molecular Compounds

The addition of small molecular compounds to EW as composite gels has been gaining increasing attention due to its easy handling. Many researchers have reported that polyphenols have a strong affinity for proteins and could improve the properties of proteins. Among the polyphenols, tea polyphenol (TP) has recently gained increasing interest in enhancing the gelling properties of EW proteins. Xue and Zhang [31] reported that the increasing addition of TP (0.01–0.05% *w*/*w*) into EW liquid could promote the formation of EW gels with high hardness and WHC by forming dense gel networks and more disulfide bonds. In addition, the appropriate addition of TP could increase the digestibility of EW gels. This is probably because the enzymatic rate of EW gels was improved during gastric digestion due to the increase of loose structures of EW proteins after adding TP [72]. In addition, TP was proposed to further enhance the performance of ultrasonic modification [49]. The hardness and WHC of TP-assisted ultrasound-modified EW gels were significantly better than those of single ultrasound-modified EW gels. This result can be ascribed to the formation of more disulfide bonds and a more stable gel conformation. Recently, Wang and Xiao [32] found that TP addition could counteract the degradation of ovalbumin brought about by a high concentration of NaOH and promote the formation of covalent bonds in the gel network structure, thus improving the mechanical properties of the EW gel. To sum up, TP can be used as an effective small molecular modifier to improve the gelling properties of EW proteins.

Genipin, a small molecule covalent crosslinker, has attracted a lot of attention in modifying the functional properties of proteins. Koyama and Kodama [33] reported that the genipin (0.9 g/L)-pretreated EW (95 g/L) protein gel (1:9 (*v*/*v*), pH 9.0, 50 °C for 8 h) exhibited up to 2.9-fold and 13% higher breaking strength and strain, respectively, as compared to the non-treated EW gel. The above results correlated with the soluble protein aggregate formed during pretreatment, which was beneficial to the formation of a finely structured gel with high breaking strength and strain. Although the genipin-pretreated EW gel had high gel strength, the EW gel turned dark blue and was unpopular.

#### 3.4.3. Biomacromolecules

Hydrophilic biomacromolecules, mainly referring to proteins and polysaccharides, are commercial gelling agents that improve the gelling properties of globular proteins. Many studies have confirmed that proteins and polysaccharides can improve the gel properties of EW proteins by affecting the gel strength, intermolecular forces, and microstructure.

Animal gelatin is naturally produced from collagen by acid- or alkali-catalyzed hydrolysis and is mostly used as a gelling, thickening, foaming, stabilizing, and water-binding agent in the food industry. The addition of gelatin to edible protein gels was suggested as a way to improve their techno-functional properties, such as gel strength and WHC. Babaei and Mohammadian [34] investigated the effects of gelatin on the gel properties of EW proteins. Results showed that the EW/gelatin composite gel containing 0.3% gelatin possessed the highest WHC and firmness indices among all samples, and that gel porosity decreased with increasing gelatin content.

Plant proteins are commonly used as an additive to improve the functional properties of other proteins because of their good gelling properties, abundant resources, and low cost. For example, the addition of soybean protein isolate (SPI) can improve the springiness and WHC of EW gels when the total protein concentration was above 0.03 g/mL due to the formation of fine microstructures through the filling and non-filling effects [73]. In addition, pumpkin-seed protein gels with a larger egg-white protein content were more elastic and more resistant to breaking force due to the formation of more homogenous and stronger microstructures [74]. Therefore, a composite protein gel system is attractive for the food industry to obtain new products with a different texture.

Dextran sulfate (DS) is a highly anionic polysaccharide derived from dextran with approximately 2.3 sulfate groups per glucosyl unit [75]. Liu and Chai [76] reported that OVA and DS can interact with each other at neutral conditions through enthalpy-driven electrostatic interactions, and their coacervates showed relatively higher thermal stability. Subsequently, Liu and Chai [35] found that the addition of DS can promote the formation of an EW/DS composite transparent gel at pH 7.0 with high gel hardness and WHC, as well as slow digestion properties. A study performed by Zhang and Yuan [77] showed that the incorporation of DS significantly inhibited the formation of large insoluble aggregates of EW proteins during heating, which is the cause for forming a transparent composite gel with a highly ordered fibrous mesh structure.

κ-carrageenan (κC) is a natural linear sulfated polysaccharide extracted from marine edible red algae and used as the thickener, stabilizer, and texturing agent in food products [78]. Mao and Huang [36] investigated the effects of κC on the structure, interaction, and rheological characteristics of OVA before and after heating. Results showed that the κC/OVA complex network was stronger than pure κ-C or OVA as a result of the strong electrostatic interactions between κ-C and OVA and the increase in the ratio of β-sheets in gel systems.

Apart from κC, gellan gum is another important gelling polysaccharide and is a negatively charged bacteria exopolysaccharide composed of repeating tetrasaccharide units (1,3-β-d-glucose,1,4-β-D-glucuronic acid,1,4-β-D-glucose,1,4-α-L-rhamnose) with one carboxyl side group [79]. Babaei and Khodaiyan [80] investigated the effect of gellan gum on the technological functionality and in vitro gastrointestinal degradation properties of EW gels at different pH values (4.0 and 7.0). The results showed that the addition of gellan gum did not drastically modify the firmness of EW gels at pH 7.0, but significantly improved the hardness and the fracture stress of the EW gels at pH 4.0. Furthermore, the enrichment of EW gels with gellan gum significantly increased their stability against in vitro gastrointestinal degradation due to the dense microstructure of binary gels.

Carboxymethylcellulose (CMC) is a non-gelling anionic polysaccharide in which the hydroxyl group on the native cellulose backbone is substituted by a carboxymethyl group [81]. CMC is usually used as a thickener and stabilizer in the food industry and exhibits high negative charge when its substitution ratio is high. A heat-set hydrogel prepared by OVA and CMC electrostatic complexes exhibited a homogeneous and dense structure and good WHC when compared with a pure OVA hydrogel at pH 4.6 [82]. This was closely related to the inhibition of the electrostatic interaction on the formation of large protein aggregates during heat treatment (90 °C, 30 min).

### 3.5. Main Differences of the Different Modification Methods

Diverse modification techniques offer the capacity to modulate both the conformational changes and aggregation dynamics of EW proteins, thereby enhancing their gelling properties. Among these approaches, physical modifications such as microwave, radio frequency, and ultrasound interventions primarily influence the aggregation state of EW proteins through a combination of thermal and mechanical effects. Alternatively, chemical methods involving glycosylation, phosphorylation, succinylation, and even redox reactions predominantly impact protein structure by modifying the side chains within the EW protein polypeptide. Biological processes, alongside the incorporation of exogenous additives, exert their influence on gel network formation through complicated mechanisms contingent upon the specific method employed. Nonetheless, it is essential to acknowledge that each modification method possesses inherent limitations, as elucidated within this section. Consequently, the pursuit of optimal outcomes necessitates the exploration of synergistic modification strategies that capitalize on the strengths of multiple approaches. This proactive pursuit is pivotal to realizing the full potential of modified EW proteins, rendering them highly suitable and effective components for diverse food product applications.

## 4. Application of EW Gels in the Food and Pharmaceutical Industries

EW proteins are traditionally used as gelling agents, gel enhancers, and quality improvers in food products such as milk egg pudding, angel cake, macaroons, and as additives in carbohydrate- and meat-based foods such as pasta, noodles, and surimi. Moreover, researchers are increasingly exploring the use of EW gel-based nutraceutical delivery systems. These systems take the form of heat-set/cold-set hydrogels, nanogels, and aerogels. They are commonly used to deliver bioactive substances such as probiotics, vitamins, functional lipids, polyphenols, and minerals. Table 2 summarizes the key impacts of EW proteins or modified EW gels.

### 4.1. Application of EW Proteins as Gelling Agents in Foods

The gel properties of protein-based hydrogels are important for regulating the texture and sensory properties of foods. EW is a useful additive because of its gelling ability and its addition could affect the quality of gelled food products. For example, the addition of EW caused significant changes in the quality characteristics and protein aggregation of oat noodles containing wheat flour and gluten [83]. The results showed that the addition of fresh EW (25%, on an oat flour weight basis) into oat noodles could significantly improve the hardness, chewiness, tensile force, and tensile distance, and promote the formation of a smooth surface, a continuous protein network, and disulfide cross-linking of proteins in the noodles. Furthermore, in addition to the improvement of texture, Rachman and Brennan [84] found that the increasing inclusion of EW proteins (0, 5, 10, and 15 g/100 g flour) in a gluten-free banana–cassava pasta decreased starch digestibility, increased protein digestibility, and improved the balance of the amino acid profile. In addition, an EW protein-fortified banana–cassava pasta had better sensory acceptance than pasta with a soy protein isolate.

Apart from pasta products, EW proteins can be used as non-meat proteins to improve the quality of meat products [85]. As reported, EW can work as a binder to bind chicken myofibrillar proteins (MPs) together and be used with soy protein isolate to synergistically improve the MP gel quality, including increased gel hardness and reduced cooking loss. In addition, the presence of NaCl in MP composite gels could lead to a stronger effect of EW on promoting MP gelation. In addition, EW has often been used as one of the major ingredients of surimi products to improve its gel texture and whiteness [86].

**Table 2:** Applications of EW proteins in the food industry.

Application Type	Materials	Object	Key Impacts	Ref.
As gelling agent	Fresh EW (0–30%)	Oat noodles	Improved textural attributes and decreased cooking loss.	[83]
	EW powder (0, 5, 10, and 15 g/100 g flour)	Banana–cassava pasta	Decreased starch digestibility, increased protein digestibility, and improved amino acid profile.	[84]
	EW powder	Chicken gel	Increased gel hardness, reduced cooking loss.	[85]
	Tea polyphenol (TP)-modified EW	Surimi gel	Significant increase in breaking force and WHC.	[87]
As gel-type delivery carriers	Cold-set EW protein/dextran sulfate hydrogels	Curcumin	Showed controlled-release properties.	[88]
	EW protein nanoparticles	Curcumin	Fibrous nanoparticles (pH 3.0) had higher curcumin loading (11.53 mg/g protein) and stability than granule nanoparticles (9.89 mg/g protein) (pH 3.8).	[89]
	EW protein nanoparticles	Linoleic acid (LA)	EW protein nanoparticles prepared by heating at 85 °C, 5 min of the 5% EW protein solution, pH 11.4 had average hydrodynamic diameters of 87 nm and LA loading capacities of 0.35 g LA/g nanoparticles.	[90]
	Acylated OVA nanogels	Curcumin	Improved encapsulation efficiency (93.63%) and sustained release of curcumin compared to non-modified OVA nanogels.	[91]
	OVA–pullulan (1:1) nanogels	Curcumin	Showed better encapsulation efficiency (88.38%), loading capacity (8.78%), and controlled release for curcumin than single OVA nanogel.	[92]
	Supercritical fluid-dried EW protein aerogel	Soybean oil	Aerogels obtained from the cold-set hydrogels had significantly higher macroporous volumes and excellent oil structuring capacities (maximal areogel-to-oil ratio of 1:15) than heat-set hydrogels (maximal areogel-to-oil ratio of 1:5).	[93]
	EW protein/CMC-Na conjugate aerogels	Soybean oil	Had a uniform structure, larger specific surface areas, higher mechanical strength, and oil holding capacities than those of the single EW aerogel.	[94]

Although EW proteins have strong gelling ability, several attempts including complexing with tea polyphenol (TP) and the Maillard reaction have been made to improve the gelling properties of EW proteins to expand their applications. Zhou and Chen [87] studied the effects of TP-modified EW (TP: 0.2%, 0.4%, 0.6%, and 0.8% *w*/*w*) on the properties of surimi. The quality of surimi gel could be markedly improved by adding TP (0.8%)-modified EW compared to the unmodified EW, which resulted in a significant increase in the breaking force and WHC of the surimi gel and the formation of a finer and more ordered network. Furthermore, SDS-PAGE indicated that TP-modified EW could increase the superior setting phenomenon of surimi gel. The analysis of FTIR spectra showed that TP modification could promote the transition of α-helices into β-sheets, possibly due to the interactions between polyphenols and C-N and N-H groups in protein polypeptide chains. Recently, the intrinsic mechanism of TP-modified EW proteins was elucidated by Xue and Zhang [31]. They suggested that the dense microstructure and improved WHC of TP-modified EW gels were correlated with the formation of more disulfide bonds among proteins, which enhanced the cross-linking degree of proteins and greatly improved the stability of the EW gel structure. This phenomenon could be due to the fact that the oxidation products of TP at high temperatures could oxidize sulfhydryl groups on the surface of the EW proteins to promote crosslinking among proteins through disulfide bonds [95]. Hence, TP-modified EW could be used as a superior gel enhancer and quality improver in surimi products compared with ordinary EW.

### 4.2. Application of EW Proteins as Gel-Type Carriers for Nutraceutical Delivery

**Figure 4:**
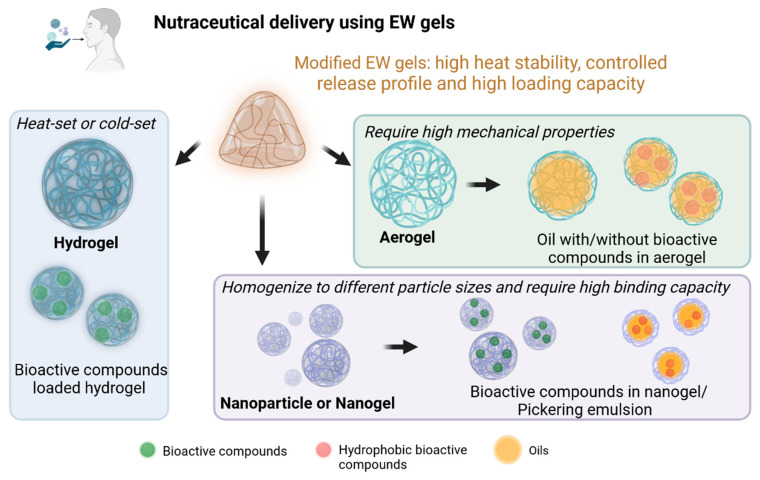
Schematic representation of three major forms of modified EW gels for nutraceutical delivery.

The development of different delivery systems with tunable physiochemical and structural properties is considered an effective approach to improve the stability and bioavailability of nutraceuticals. Chicken EW and its component proteins are versatile and ideal materials to fabricate delivery systems due to their abundance, safety, and amphiphilicity. EW gels can have three main forms as nutraceutical delivery systems (Figure 4). Using the different modification strategies, the functionality of EW protein-based delivery systems can be tailored for specific applications. Specifically, compared with native EW proteins, EW proteins derived from hydrogels, nanoparticles or nanogels, and aerogels have their particular advantages, such as high heat stability, controlled release, and high loading capacity.

### 4.3. EW Hydrogels as Delivery Carriers

Hydrogels are an important class of nutraceutical delivery systems with benefits such as pH-responsive delivery and controlled release. EW protein hydrogels can be classified into heat-set hydrogels and cold-set hydrogels, depending on their gelation methods. A heat-set hydrogel is restricted to heat-insensitive bioactive molecules. Compared to heat-set hydrogels, cold-set hydrogels have been considered as carriers with more potential to produce healthy foods, as they allow the incorporation of thermal-sensitive bioactive ingredients into a cold aggregated protein solution before the triggering of gelation by the addition of acidulant agents, salts, or enzymes. Alavi and Emam-Djomeh [96] developed a method to prepare cold-set EW hydrogels by preheating EW proteins (65–90 °C for 30 min) at a highly alkaline pH (about 11.3) and acid-induced gelation (pH 4.5). They found that preheating EW proteins at a highly alkaline pH could form a thermally aggregated EW protein with numerous disulfide bonds, hydrophobic interactions, and a network of β-sheet structures. Hence, the corresponding acid-induced gels exhibited a weak dependence on storage modulus (G′) with frequency. As mentioned in Section 3.4, hydrogels assembled from EW proteins and other ingredients may provide superior properties to single EW protein-based hydrogels. For example, Liu and Chai [88] designed an acid-induced cold-set composite hydrogel of EW proteins/dextran sulfate with improved mechanic properties for encapsulating curcumin (Cur). In their study, DS and MTGase were used to control the aggregation and gelation behavior of EW proteins at the preheating step and gelation step, respectively. The resultant cold-set hydrogel with high gel hardness, storage modulus, and WHC can effectively slow down the release rate of Cur in gastrointestinal digestion, therefore exhibiting great potential as a delivery carrier with controlled release properties. The above results demonstrated that the hydrogel with higher strength and storage modulus has better sustained release.

### 4.4. EW Nanoparticles or Nanogels as Delivery Carriers

Homogenizing the formed hydrogel is a commonly applied method to prepare EW-protein nanoparticles or nanogels. The morphology of these nanoparticles is strongly dependent on the environmental conditions during the heating process. As reported by Chang and Niu [97], the morphology of protein nanoparticles prepared by acid heat gelation depends on ionic strength and protein concentration. At pH 3.0, the EW protein particles formed a fine fibrous structure. They would transform from a fibrous structure to a granular structure with increasing protein concentrations or the addition of salt, whereas EW proteins can only form granular microparticles at pH 3.8 under various protein concentrations. Afterwards, EW nanoparticles prepared using this acid heat gelation method were applied to encapsulate Cur. They showed that EW nanoparticles can significantly reduce the degradation of Cur when stored at 4 °C for 3 days [89]. Meanwhile, the fibrous EW nanoparticles prepared at pH 3.0 had higher Cur loading than those of granule EW nanoparticles prepared at pH 3.8. This result can be ascribed to the stronger Cur-binding capacity of highly hydrophobic fibrous nanoparticles.

In addition to the acid heat gelation method, the nanoparticles of hydrophobic compounds can be obtained by the controlled heating of EW protein dispersions at an alkaline pH. Sponton and Perez [98] evaluated the effects of heating time (0–20 min) and solution pH (9.6–11.4) on particle size, surface hydrophobicity, and binding capacity for linoleic acid (LA). Results showed that pH exerted the most critical effects on the particle size and LA binding capacity of EW protein nanoparticles. In a subsequent work, Sponton and Perez [99] investigated the impact of EW protein concentration on the LA binding capacity and particle size of heat-induced EW protein nanoparticles. Results indicated that the LA binding capacity of EW protein nanoparticles was almost unaffected by protein concentration, although both turbidity and average particle diameter increased linearly with protein concentration. Subsequently, Sponton and Perez [90] prepared EW protein nanoparticles using industrial liquid EW in a batch process, including dilution, pH adjustment, centrifugation, and heating. Their results indicated that this batch process would be reproducible in terms of particle size and LA binding ability, which would allow the production of EW protein nanoparticles to be scaled up for delivering hydrophobic bioactive substances.

Nanoparticles, nanogels, or Pickering emulsions made by EW proteins alone may possess weak mechanical strength or undergo rapid disruption during digestion, limiting their application in the delivery of bioactive substances. Hu and Batool [91] fabricated acylated OVA nanogels via succinic anhydride modification (20%, *w*/*w*, protein base) and heat-induced self-assembly (90 °C, 30 min) for the delivery of Cur. They found that acylated OVA nanogels had a smaller average hydrodynamic diameter (155.73 nm) and higher stability due to enhanced negative surface charge (−24.3 mV). In addition, the obtained acylated OVA nanogels exhibited improved encapsulation efficiency and sustained release of Cur compared to non-modified OVA nanogels. Complexing with polysaccharides is another feasible method to further improve the gelling properties of OVA. Zeng and Zeng [92] fabricated an OVA–pullulan (1:1) nanogel through the Maillard reaction combined with heat treatment. This composite nanogel (around 190 nm) exhibited excellent storage stability and better encapsulation efficiency (88.38%), loading capacity (8.78%), and controlled release for Cur than a single OVA nanogel. These results could be ascribed to the good cross-linking between OVA and pullulan through heat-denatured gelation, as well as the hydrophilic polysaccharide chains at the exterior of nanogels.

### 4.5. EW Aerogels as Delivery Carriers

An aerogel is a highly porous material with a large internal specific surface area and has been used as a delivery system to improve the stability and pharmacokinetics properties of bioactive substances. Recently, supercritical carbon dioxide dried superlight microporous aerogels from cold-set EW protein hydrogels were developed by Alavi and Ciftci [93], in which the cold-set hydrogels were prepared through preheating an EW solution at a highly alkaline pH and acid gelation by GDL. The aerogels obtained from the cold-set hydrogels had lower density and significantly higher macroporous volume than heat-set hydrogels, due to the lower biopolymer concentration and less shrinkage of their precursor hydrogels.

However, the network structure and strength of single EW protein gels need to meet current demands for the formation of protein-based aerogels. For desirable oil-loading matrices, EW aerogels are required to achieve good mechanical properties and loading efficiency to broaden their applicability. To improve the mechanical strength and oil-loading performances of EW aerogels, Tang and Jiang [94] investigated the effect of sodium carboxymethylcellulose (CMC-Na) glycosylation on the physicochemical properties of EW proteins, as well as the microstructure, mechanical properties, pore parameters, and oil-carrying properties of the corresponding aerogels. The slope of the stress-compression curve can be employed to assess the stiffness of the aerogels. The greater the slope, the greater the stiffness of the aerogel. The grafted aerogel samples prepared by EW protein/CMC-Na conjugates with a grafting degree of 8.35% by dry heating at 60 °C for 12 h exhibited higher stiffness than the single EW protein aerogel. Furthermore, this aerogel achieved an oil absorption capacity of 5.46 g/g aerogel and an oil holding capacity of 31.95%, which were nearly 1.7 times higher than those of EW aerogel. The findings of this study indicate that modified EW aerogels with enhanced stiffness exhibited superior oil absorption capacities and have the potential to serve as carriers for encapsulating fat-soluble active substances.

### 4.6. Importance of Processing Control and Storage Conditions

As mentioned above, processing parameters such as pH, gelation methods, and the grafting degree of functional groups or polysaccharides significantly affect the performance of EW hydrogels, nanogels, and aerogels. The properties of EW hydrogels have a decisive role in the performance of nanogels and aerogels. The properties of a nanogel depend on the initial state of its hydrogels or aggregation particles, such as particle size, charge, and hydrophobicity, whilst aerogels rely more on the corresponding gel strength of hydrogels. This is because a high-strength hydrogel generally has a homogeneous microscopic network. When this hydrogel is subjected to the preparation process of an aerogel, an aerogel with a large pore-specific surface area and a stable rigid structure will be formed. Therefore, optimizing the processing parameters is indispensable in preparing EW hydrogels, nanogels, and aerogels to maximize their applicability. When these EW gels are applied for nutraceutical delivery, storage conditions must be considered. Except for aerogels, hydrogels and nanogels are waterish and perishable. When hydrogels and nanogels are used to load nutraceuticals, how to choose the appropriate sterilization, preservation, and drying methods needs to be further studied.

## 5. Research Gaps between Modified EW Gels and Their Applications

Foods are multicomponent systems, primarily comprising proteins, starches/gums, and lipids. In this context, native or modified EW proteins play a crucial role, engaging in interactions with these essential food constituents. Prior research has showcased enhanced gelling properties of EW proteins through modifications using diverse techniques, including physical, chemical, biological, and exogenous additives, often in combination. Nevertheless, investigations targeting the utilization of these modified EW gels to enhance texture and water retention in food systems remain to be investigated in detail. Consequently, a pressing need arises for deeper explorations into the interactions between modified EW gels and other protein, starch/gum, and lipid components derived from food sources. By exploring these complex interactions, the versatility of modified EW gels can be fine-tuned, rendering them better suited for specific or multifaceted product applications. Moreover, a major challenge revolves around finite protein aggregation or the type and strength of gel networks within EW gels and their modified counterparts, including nanoparticles, nanogels, and aerogels, particularly when employed as delivery carriers. In response, recent research has indicated that the combined use of modification methods or the creation of binary/multicomponent composite systems could serve as indispensable avenues to optimize the practical utility of EW gels. As such, the fusion of multiple technologies or materials emerges as an effective strategy to bridge existing gaps between modified EW gels and their potential applications within the food industry. In essence, combining different methods strategically can help boost the significance of these modified gels in the fields of food and medicine.

## 6. Concluding Remarks and Future Perspectives

In summary, chicken egg white (EW) stands as a valuable natural source of functional and bioactive food proteins, with ovalbumin playing a pivotal role in governing gelling properties that crucially influence texture and water-holding capacity. The gelation mechanisms are related to the unfolding and aggregation of EW proteins, driven by complex molecular interactions encompassing hydrogen bonding, hydrophobic interactions, and electrostatic forces. To enhance the gelling properties, a range of modification methods has been utilized, including physical, chemical, biological, and exogenous component addition techniques. These strategies offer distinct advantages, extending the potential applications of EW gels into conventional foods and nutraceutical supplements. However, given the interplay between modified EW proteins and food or drug constituents, a singular modification approach may fall short of ensuring adaptability across diverse scenarios. The insights into developing modification strategies for EW gels play a key role in realizing the potential of EW proteins across a spectrum of applications. Moreover, beyond their role in gelled food products and nutraceutical carriers, EW gels hold promise as versatile emulsifying and foaming agents. Recent explorations into emulsions and innovative aerated products underscore their multifunctional capabilities, an area ripe for further investigation to reveal the breadth of their potential.

## Figures and Tables

**Table 1:** Action mechanisms, advantages, and drawbacks of the main physical, chemical, biological, and exogenous addition methods.

Types	Methods/Materials	Processing Conditions	Outcome	Action Mechanisms	Advantages	Drawbacks	Ref.
Physical	Hot room dry heating	75 °C and 65% humidity for 15 d	Increased gel hardness by 2.10-fold	Appropriate heat and mechanical denaturation, changed protein structure and aggregation state	Mature and stable technology	Long-time, high-energy cost	[21]
	Radiofrequency heating	80 °C for 5 min	Increased gel Firmness by 48.6%		Short time	High temperature	[22]
	Microwave (MW)	100 °C for 15, 30, and 60 min	Increased gel firmness by 3–5 fold		Short time	High temperature	[23]
	Ultrasound (US)	375 W/L (dual frequency: 20/40 kHz)	Decreased the mobility of free water		Low temperature, short time	Only for liquid	[24]
	US and MW heating	57 °C for 2 min with US power of 700 W	Increased gel strength from 261 g to 338 g		Uniform, short time	Complicated instrument	[25]
Chemical	Glycosylation or succinylation	60 °C for 3 h D-xylose: EW powder (1:2, *w*/*w*)	Increased gel hardness from 80 to 140 g	Introduce new functional groups	Increased hydrophilicity and changed structures	Additional non-egg ingredients	[26]
Biological	Transglutaminase	85 °C for 30 min and then treated by MTGase at pH 7.5.	Increased hardness from 275.6 g to 419.3 g	Form additional intra- or inter-molecular bond	Suitable for preparing cold-set hydrogels	Not ideal for native EW proteins	[15,27]
	Lactic bacteria fermentation	37 °C for 7 h by *Lactiplantibacillus plantarum*.	Decreased hardness by about 1/7	Involve acidification and hydrolysis	Improve gel appearance and decrease pH value	Partial consumption of EW proteins	[28]
Exogenous additives	Salts (NaCl, KCl, Na_2_SO_4_, NaOH, and polyphophate)	e.g., sodium tripolyphosphate 0.45%, *w*/*v*	Increased hardness to 768.17 g	Change the charges and intermolecule forces of proteins	Simple operation	Changed taste, hampered by regulations	[19,29,30]
	Small molecular compounds (Tea polyphenol and genipin)	The EW liquid containing tea polyphenol (0.01–0.05% *w*/*w*) heated at 85 °C for 20 min.	Hardness ranged from 971.43 g to 1261.63 g	Change the structure and cross-linking properties of EW proteins	Simple operation, increase the digestibility and rigidity of EW gels	Changes the sensory attributes of EW gels	[31,32,33]
	Biomacromolecules (Gelatin, dextran sulfate, and carrageena)	Depends on their types (e.g., dextran sulfate)	Increased hardness from about 580 g to 700 g	Form composite gel systems by non-covalent interactions	Simple operation	Additional non-egg proteins or polysaccharides	[34,35,36]

## Data Availability

No new data were created or analyzed in this study. Data sharing is not applicable to this article.
